# Lord’s Paradox and two network meta-analysis models

**DOI:** 10.1017/rsm.2025.10036

**Published:** 2025-09-18

**Authors:** Yu-Kang Tu, James S. Hodges

**Affiliations:** 1 Institute of Health Data Analytics & Statistics, College of Public Health, National Taiwan University, Taipei, Taiwan; 2 Health Data Research Center, National Taiwan University, Taipei, Taiwan; 3 Division of Biostatistics and Health Data Science, School of Public Health, University of Minnesota, Minneapolis, MN, USA

**Keywords:** network meta-analysis, contrast-based model, baseline model, Lord’s paradox, directly acyclic graph

## Abstract

The contrast-based model (CBM) is the most popular network meta-analysis (NMA) method, although alternative approaches, e.g., the baseline model (BM), have been proposed but seldom used. This article aims to illuminate the difference between the CBM and BM and explores when they produce different results. These models differ in key assumptions: The CBM assumes treatment contrasts are exchangeable across trials and models the reference (baseline) treatment’s outcome levels as fixed effects, while the BM further assumes that the baseline treatment’s outcome levels are exchangeable across trials and treats them as random effects. We show algebraically and graphically that the difference between the CBM and BM is analogous to the difference between the two analyses in a statistical conundrum called Lord’s Paradox, in which the *t*-test and analysis of covariance (ANCOVA) yield conflicting conclusions about the group difference in weight gain. We show that this conflict arises because the *t*-test compares the *observed* weight change, whereas ANCOVA compares an *adjusted* weight change. In NMA, analogously, the CBM compares observed treatment contrasts, while the BM compares adjusted treatment contrasts. We demonstrate how the difference in modeling baseline effects can cause the CBM and BM to give different results. The analogy of Lord’s Paradox provides insights into the different assumptions of the CBM and BM regarding the relationship between baseline effects and treatment contrasts. When these two models produce substantially different results, it may indicate a violation of the transitivity assumption. Therefore, we should be cautious in interpreting the results from either model.

## Highlights

### What is already known?

The CBM for NMA assumes treatment contrasts are exchangeable across the included studies, while the BM further assumes the baseline (or reference) treatment’s outcome level is also exchangeable across trials. A recent study showed that these two models may yield different results when the baseline risks vary across different designs of studies that compared different sets of treatments.

### What is new?

We show that a key distinction between these two NMA models is similar to Lord’s Paradox, a statistical conundrum about whether a *t*-test or ANCOVA should be used to compare two groups according to a change in the outcome. We show that the CBM uses the observed treatment contrasts as the outcome in the analysis, while the BM uses adjusted treatment contrasts as the outcome. Alternatively, the CBM estimates the unconditional differences between one treatment and the baseline treatment, while the BM estimates the conditional difference by adjusting for the baseline effects.

### Potential impact for RSM readers

The two NMA models make different assumptions about the relationship between treatment contrasts and the reference treatment effects. These differences in statistical assumptions reflect different perspectives on how the data were generated and how treatments impact patients. Therefore, choosing the appropriate model should depend on evaluating the validity of these assumptions in real-world scenarios.

## 1 Introduction

Network meta-analysis (NMA) combines direct and indirect evidence to compare the benefits and harms of multiple treatments.^1^ ^–^ ^3^ Several approaches have been proposed to estimate relative effects between treatments; differences in their model specifications reflect different assumptions about how treatments should be compared.^4^ ^,^ ^5^ The contrast-based model (CBM), also called the Lu and Ades model, uses the difference in outcome between a pair of treatments in a given study, i.e., a treatment contrast, as the unit of analysis, assuming that a given treatment contrast is exchangeable across studies.^6^ ^,^ ^7^ The baseline model (BM) further assumes that the reference treatment’s outcome levels are exchangeable across studies.^1^ ^,^ ^5^ Although these two models are closely related, there has been debate as to which should be preferred.^4^ ^–^ ^6^ ^,^ ^8^ ^,^ ^9^

White et al.^1^ ^,^ ^5^ discussed when the CBM and BMs yield different results. We aim to illuminate a key difference between these two models and to show that this difference is analogous to Lord’s Paradox, a conundrum about whether a *t*-test or analysis of covariance (ANCOVA) should be used to compare two groups according to a change in an outcome.^10^ ^,^ ^11^

Our article is organized as follows. First, we give a brief overview of the CBM and BM and their assumptions. We then describe Lord’s Paradox and how the paradox occurs, after which we discuss how the assumptions of the two NMA models are related to Lord’s Paradox, using directed acyclic graphs (DAGs) to illustrate the relation. Finally, we discuss how Lord’s Paradox can illuminate the differences between the two NMA models.

## 2 NMA models and their statistical assumptions

Both NMA models were initially described within the Bayesian statistical framework.^1^ ^,^ ^4^ ^,^ ^12^ For simplicity, we use the frequentist framework.

### 2.1 Contrast-based model

The CBM assumes a given treatment contrast is exchangeable across studies, as standard pairwise meta-analysis does. The CBM can be written as

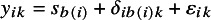



(Model 1)

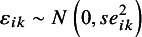




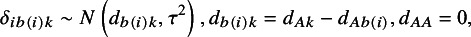

where 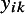 is treatment *k*’s response in study *i* with standard error 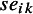 ; 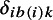 is treatment *k*’s trial-specific effect relative to trial *i*’s baseline treatment 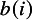 ; 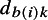 is the average of the contrast between *k* and 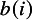 ; 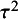 , a variance, describes heterogeneity between trials in this contrast, and 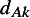 is the difference between the average of treatment *k* and a reference treatment *A*. The heterogeneity 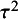 is usually assumed identical for all treatment contrasts, so their covariances are 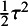 . For binary data, 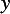 is usually the log odds or log risk, so 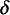 and 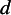 are the log odds ratio or log risk ratio between two treatments. For continuous data, 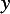 is the outcome or change in outcome from the start of observation, so 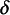 and 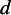 are the mean difference in the outcome between two treatment groups. For either type of data, 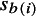 is a fixed-effect parameter specific to study *i*, often interpreted as a study effect, while 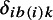 is a random effect following a normal distribution, implying that it is exchangeable across studies.

### 2.2 Baseline model

The BM differs from the CBM in that the reference treatment’s level is modeled as a random effect with a separate draw from a normal distribution for each study. If we designate treatment A as the reference treatment, regardless of whether it was included in every study, we can write the BM (in arm-based form) as^12^


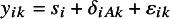



(Model 2)

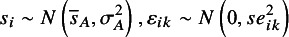




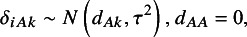

where 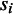 is the (possibly hypothetical) true response for treatment *A* in study *i*, and 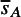 and 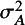 are the mean and variance across studies of this true response; 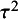 is assumed identical for all treatment contrasts, so their covariances are 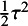 . The random effects 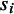 and 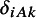 are assumed independent. (Model-2 is equivalent to Model 3 in the article by White et al. therein called the CBM with random study intercepts.)^5^ Different specifications of this model have been published. As specified in Model 2, the analysis results do not depend on the choice of reference treatment; as specified in White et al.^5^, the results do depend on the choice of reference treatment. The Supplementary Material gives more details. Other parameters are as in the CBM. Thus, the baseline and CBMs differ in that 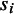 is a draw from a random effect in the former but a fixed effect in the latter.

### 2.3 Statistical assumptions of these NMA models

The study effect 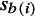 in the CBM, a fixed effect, is the response of study *i*’s control group to the baseline treatment; the baseline treatment response is specific to each study and is not assumed exchangeable across studies. Rather, the difference in responses between two treatment groups—the treatment *contrast* or relative effect—is exchangeable.

This assumption that treatment contrasts are exchangeable further implies no association between study *i*’s baseline effect 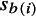 and study *i*’s relative effect 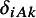 . Suppose all studies include the reference treatment *A*. The assumption is that if *A* has a large or small absolute effect in study *i*, this has no relation to the *relative* effect between *A* and the other treatments in study *i*. Even if the included studies come from different populations with different distributions of treatment effect modifiers, such as age, the relative treatment effects between *A* and other treatments in different studies remain similar by assumption, although the absolute effect of *A* may vary from study to study with differences in age.

The BM assumes baseline treatment *A*’s effect is exchangeable across studies, a random effect with a normal distribution. When a study’s patients are randomly assigned to *A* and other treatments, that study’s other treatments are thus randomly sampled from the same population, so the BM implicitly assumes all treatment effects are exchangeable across studies.^5^ We revisit this later when we discuss the selection of a baseline treatment.

## 3 Lord’s Paradox

We may gain insight into the differences between the CBM and BM from the literature about Lord’s Paradox.^10^ ^,^ ^11^ This literature is vast^13^ ^–^ ^16^; we do not provide a comprehensive review.

In FM Lord’s original article, a university studied the effect on students’ body weight of the diet provided in the university dining halls. The study also asked whether males and females differed in the effects of the diet. Each student’s weight was measured upon their arrival in September and again the following June. Two statisticians analyzed the data independently.

### 3.1 A numerical example

For our demonstration, we simulated body weights of 100 female and 100 male students. Table 1 summarizes the simulated data.

**Table 1:** Summary statistics for the hypothetical body weight data with 100 male and 100 female students

	Gender	
	Females	Males
*N*	100	100
bw1	56.21 (9.50)	70.90 (9.13)
bw2	55.75 (9.61)	70.08 (9.10)
cw	−0.46 (6.60)	−0.83 (6.17)

*Note:* bw1, body weight measured at baseline; bw2, body weight measured at follow-up; cw, change in weight.

The first statistician calculates the mean weights of female students at the beginning and end of the year (56.20 kg and 55.75 kg, respectively) and finds the change (−0.46 kg) very small. The mean weights of male students at the beginning (70.90 kg) and end of the year (70.08 kg) are also similar, and the change (−0.83 kg) is also small. She compares weight change between females and males using a *t*-test, obtains a *p*-value of 0.68, and concludes that females and males did not differ in weight change. This t-test can be written as a linear regression model:
(1)

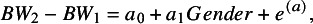

 where 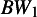 and 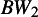 are body weights measured at baseline and follow-up, respectively, and *Gender* is a dummy variable with females coded 0 and males 1. The estimate of 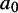 is −0.46 kg, the mean weight change in females, and 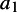 is −0.37 kg, suggesting no difference between sexes in mean weight change.

The second statistician notices that males are larger than females at baseline on average (70.9 kg vs 56.2 kg), so she uses ANCOVA to adjust for the baseline difference in body weight. The ANCOVA model can also be written as a linear regression:
(2)

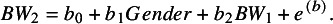



**Figure 1:**
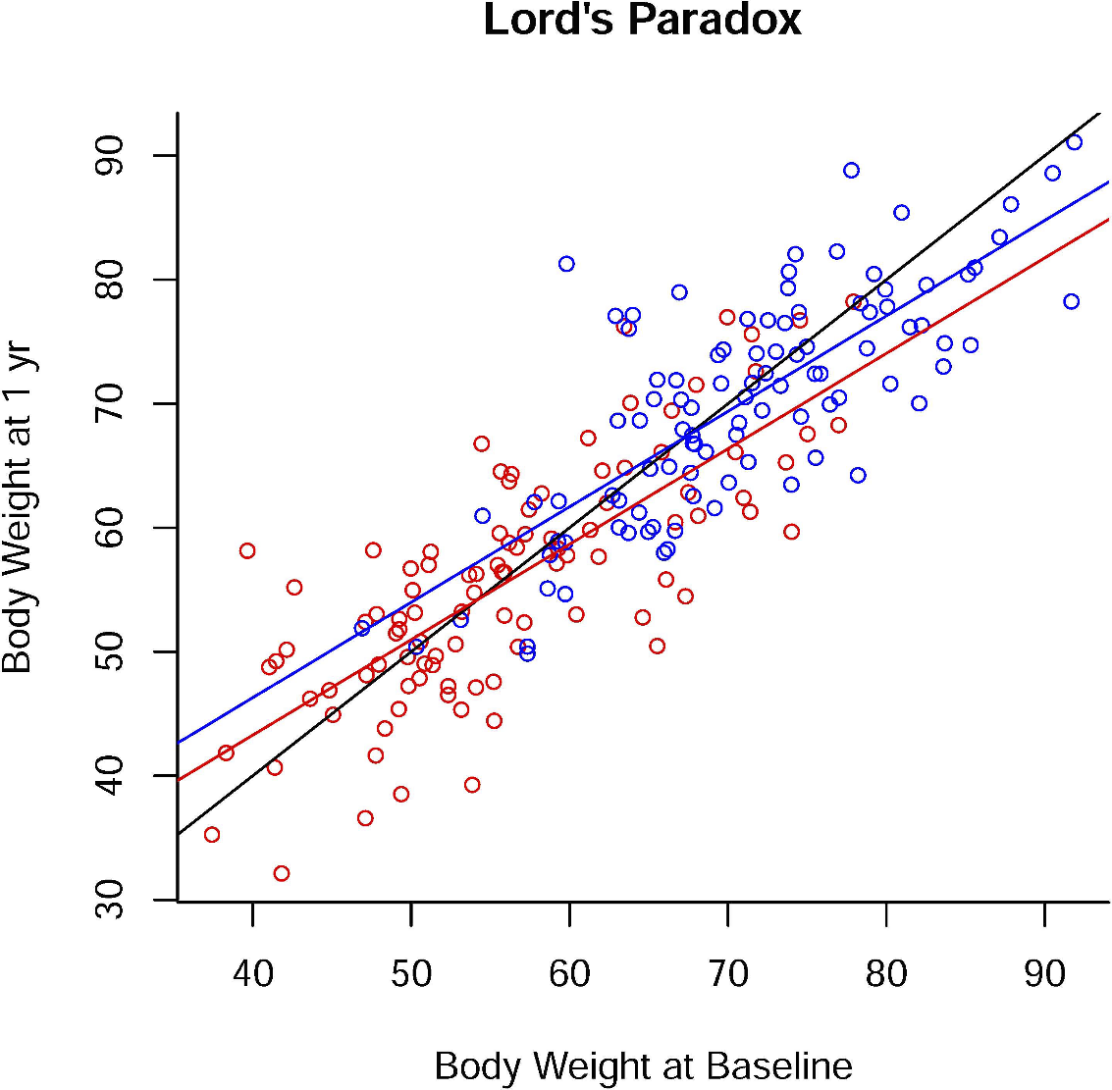
Scatterplot of the hypothetical data with 100 male (blue circles) and 100 female (red circles) students. The blue and red solid lines are the fitted regression lines for male and female students, respectively. The black solid line has an intercept of zero and a slope of 1.

The estimate of 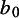 is 12.49 kg, the estimated mean body weight at the end of the year for females whose baseline body weight is zero, so 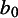 has no practical meaning. The estimate of 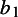 is 3.02 kg, the estimated difference in follow-up body weight between females and males with the same baseline body weight. The estimate of 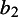 is 0.77, the estimated difference in follow-up body weight between students of a given gender whose baseline body weights differ by 1 kg. Because 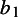 is statistically significant, the second statistician concludes that, on average, males gain 3 kg more than females. The horizontal and vertical axes of Figure 1’s scatterplot are the baseline and follow-up body weights, respectively. Red and blue circles represent females and males. Red and blue lines are fitted parallel regression lines for females and males, respectively, both with slope 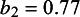 . Their intercepts differ by 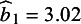 .

### 3.2 How does Lord’s Paradox happen?

When the difference between males and females in mean body weight change is small, the regression coefficient 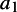 in Equation (1) will be close to 0. However, 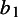 in Equation (2) equals 0 only under very special conditions.

Equation (1) can be rearranged as
(3)

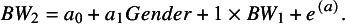



The *t*-test is thus a linear regression in which 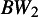 is regressed on 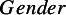 and 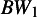 with 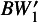 s coefficient constrained to 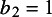 , represented by Figure 1’s black line. When 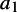 is close to 0 as in our example, the fitted black lines for males and females are indistinguishable. Comparing Equations (3) and (2), we see that Lord’s Paradox arises when 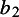 is substantially less than 1 in Equation (2), in which case the two statisticians will give different conclusions.

We can rearrange Equation (2) as
(4)

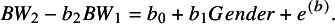



Comparing Equations (1) and (4), Lord’s Paradox can be interpreted as follows: The first statistician regresses the *observed* weight change 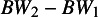 on 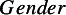 , while the second regresses the *adjusted* weight change 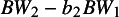 on 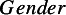 . If 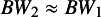 , as in our example, then 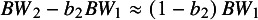 . Because both males and females have tiny observed weight changes, 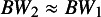 for both genders, so the two genders show a negligible difference in weight change. Thus, the first statistician finds no difference in weight change between females and males. In contrast, males have greater 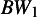 than females, so males have greater adjusted weight change. In our example, the average adjusted weight changes for males and females are 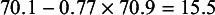 and 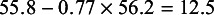 , respectively, so the difference is about 3 kg, as the ANCOVA estimates.

### 3.3 An alternative formulation of Lord’s Paradox

Alternatively, we can consider the difference between the first and second statisticians as arising from their differing assumptions about the relationship between change score and baseline body weight. The first statistician assumes no relationship between weight change and baseline body weight, because Equation (1) can be expressed as
(5)

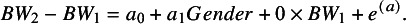



The coefficient for 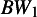 is 0, so Equation (1) assumes weight change is not related to baseline body weight. Thus, although males have greater body weight than females, on average, that plays no role in comparing their weight changes.

The second statistician assumes there *is* a relationship between weight change and baseline weight, as seen when Equation (2) is reexpressed as
(6)

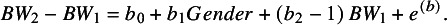



The two statisticians reach the same conclusion if 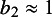 . Because of random errors in weight measurements and natural variation in body weight *within* each student, 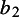 is likely to be rather less than 1.^14^ ^,^ ^17^ This is what FM Lord tried to demonstrate in his short article.^10^

## 4 The relation between NMA models and Lord’s Paradox

We use an NMA with treatments X, Y, and Z to show how Lord’s Paradox illuminates the differences between the two NMA models. Following White et al.^1^ ^,^ ^5^, our NMA includes only trials comparing two treatments, one of which is treatment X. Thus, this NMA includes only two types of trials, also called designs in the NMA literature^18^: one comparing X with Y (design XY) and the other comparing X with Z (design XZ). X is each trial’s baseline treatment and also the network’s reference treatment, so it is treatment “A” in Model-1 (the CB model) and Model-2 (the BM) above. We wish to know whether Z’s effect differs from Y’s.

The CBM for this NMA can be expressed (in contrast-based form) as a regression model:

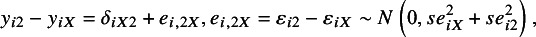

 where group 2 is either Y or Z; group 1 is always X so the subscript “1” has been replaced by X. Model 1’s trial-specific effect of treatment *k* relative to trial *i*’s baseline treatment 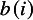 , 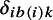 , can be expressed as

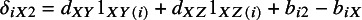

 where 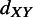 and 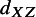 are as in Model 1; 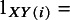 1 when study *i* has design XY and 0 when study *i* has design XZ; 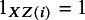 when study *i* has design XZ and 0 when study *i* has design XY; and 
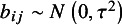
 captures heterogeneity. The CBM is then
(7)

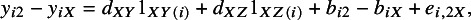

 where the subscript 2 for 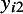 represents each study’s second treatment group, Y or Z. We can add 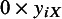 to Equation (7), so it becomes clear that the CBM assumes no relationship between the treatment contrast and the baseline treatment:
(8)

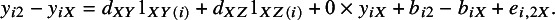



Equation (8) can be further rearranged by moving 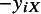 to the right side, yielding:
(9)

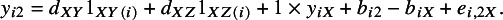



The CBM can thus be viewed as regressing the test treatment level on the reference treatment level, with the regression coefficient fixed at 1. In all these ways, the CBM’s analysis is like the first statistician’s analysis in Lord’s Paradox.

Now consider the BM for this NMA, Model 2. Suppose we have an estimate 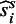 for 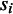 . Such an estimate using Model 2, a mixed linear model, will be shrunk (hence the superscript) toward 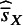 , an estimate of 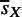 , the mean of 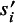 s distribution, so 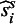 can be written

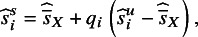

 where 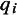 is the shrinkage factor and 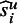 is the unshrunk estimate of 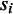 from a CBM fit, where the baseline treatment is a fixed effect. Each study and arm has its own sampling-error variance (usually treated as known), so 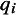 is a complex function of the heterogeneity 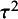 and reference-group random-effect variance 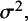 but it is easy to show that as 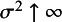 i.e., as the BM tends toward the CBM, 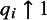 .

Now 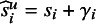 and 
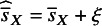
 , where 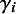 and 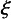 have mean zero because the respective estimates are unbiased. Therefore,

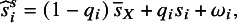

 where 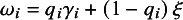 has mean 0. If we replace the hypothetical true response for treatment 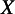 for study *i*, 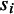 , in Model 2 with its estimate, 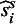 , then

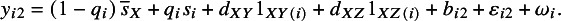

 where the sum of the last three terms has a mean of zero.

Now 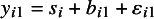 , so
(10)

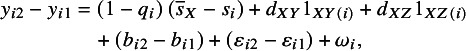

where the second line of Equation (10) has a mean of zero.

Equation (10) can be rewritten in ways that are analogous to Equations (4) and (6). First, recall that 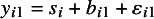 . Gather these items from Equation (10)’s right side to give
(11)

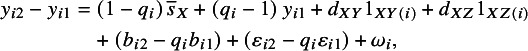

where Equation (11)’s second line has mean zero. Equation (11) and Equation (6) have the same form: The BM implicitly assumes the study-specific treatment contrast and baseline effect are associated, while the CBM assumes they are independent.

Finally, we can move 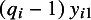 to the left-hand side of Equation (11), giving
(12)

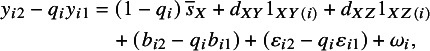

where the second line of Equation (12) has mean zero. Equation (12) and Equation (4) have the same form: The BM, in effect, has as its dependent variable an *adjusted* difference between the non-reference and reference treatments, where the adjustment depends on the shrinkage induced by the random effect used to model the reference treatment.

We make two comments about the BM. First, the shrinkage factor 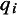 has the same role that 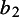 has in Lord’s Paradox in Equations (4) and (6), but 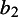 and 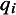 differ in other ways: 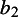 is the same for all students in Lord’s example, while 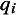 takes different values for different studies 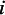 , and 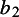 is estimated by fitting the regression in Equation (4), while the 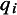 depend on the data indirectly through the study sample sizes, the estimates 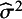 and 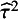 , and the 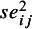 . The 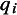 will be close to zero only for very small studies. Second, 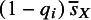 is present in Equations (11) and (12) but has no analog in Equations (4) and (6). From Equation (11), biases in the BM’s estimates of treatment effects are a function of these terms.

### 4.1 NMA models and the two statisticians in Lord’s Paradox

Comparing Equations (5) and (8) and Equations (6) and (12), the CBM is the first statistician, while the BM is the second statistician. Each student has two measurements of body weight, as each trial has two treatments. Each student’s weight change corresponds to the treatment contrast in each trial. Males and females correspond to designs XZ and XY, respectively. The two statisticians’ approaches yield different results when males have larger baseline body weight; the two approaches to NMA may yield different results when the effects of baseline (and reference) treatment X differ between the two trial designs.

## 5 Graphical comparisons of NMA models

**Figure 2:**
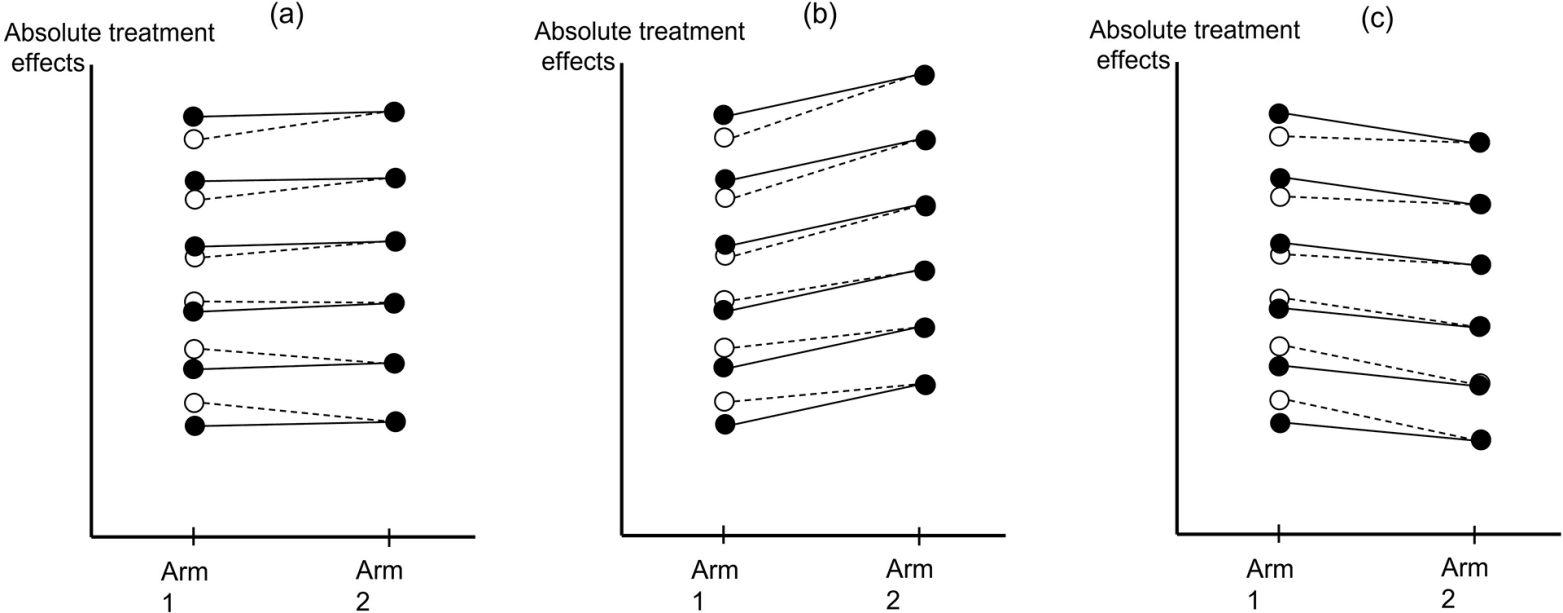
Line plots for comparing contrast-based and baseline models. The two filled circles connected with a solid line represent a trial comparing two treatments, Arms 1 and 2. The open circles are the shrunken estimates of Arm 1 given by the baseline model; these open circles are “shrunk” closer to the average effect of Arm 1. The horizontal axis is the treatment arms, and the vertical axis is the absolute treatment effects. In (a), the two arms in each of the six trials have the same treatment effects. In (b), Arm 2 is better than Arm 1, and in (c), Arm 1 is better than Arm 2. The difference between the two arms is identical in every trial.

Figure 2 graphically explains the difference between the two models’ results in different scenarios. In terms of the preceding development for the BM, Figure 2 connects most directly to Equation (10). In Figure 2, two filled circles connected with a solid line represent a trial comparing two treatments, Arms 1 and 2. The horizontal axis is the treatment arms, and the vertical axis is the absolute treatment effects. In Figure 2a, the two arms in each of the six trials have the same outcome levels. In Figures 2b and 2c, the two arms have different outcome levels, but the difference between the two arms is identical in all trials. The open circles are the BM’s shrunken estimates for Arm 1, which are “shrunk” toward Arm 1’s average level.

Suppose we include two types of trials in the analysis: design XY and design XZ. X is Arm 1, each study’s baseline treatment, and the network’s reference treatment, and we aim to know whether Z differs from Y. In the CBM, the difference between the two arms’ outcomes in each trial is the difference between the values of each trial’s two filled circles. In the BM, however, the difference in treatment levels is the difference between the open circle on the left and the solid circle on the right, connected by the dashed line. Because X’s level is modeled as a random effect, for trials with above-average levels of X, their estimated levels of X are shrunk downward, and for trials with below-average X, their estimated X are shrunk upward.

When designs XY and XZ have the same distribution of X, e.g., each design includes all six trials in Figure 2, Y and Z do not differ using the CBM or BM. But if the top three trials have design XZ and the bottom three trials have design XY, the two models give different results. In Figure 2a, the CBM finds no difference between Z and Y, but the BM finds Z is better than Y (because Z is better than X in design XZ, and X is better than Y in design XY). In Figure 2b, the CBM finds that both Z and Y are better than X, and Z and Y do not differ. The BM, however, finds that both Z and Y are better than X, but Z is also better than Y. In Figure 2c, the CBM finds that X is better than Z and Y, and again Z and Y do not differ. The BM now finds that X is slightly better than Z but much better than Y, so Z is better than Y.

## 6 Directed acyclic graphs for Lord’s Paradox and NMA models

**Figure 3:**
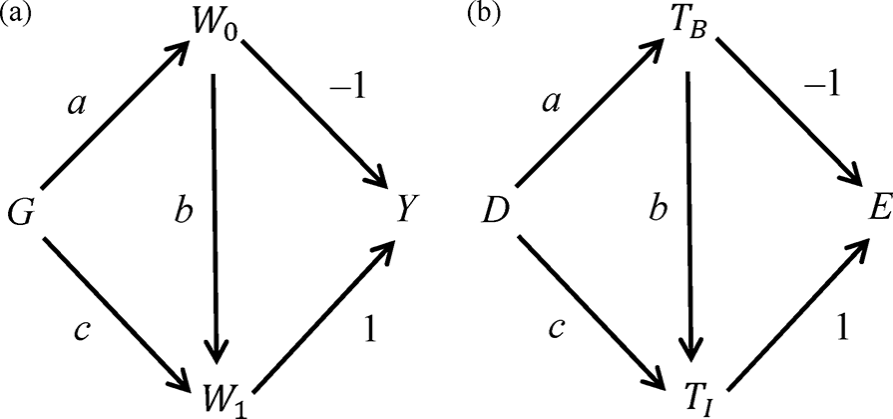
*Directed acyclic graphs for (a) Lord’s Paradox:* 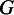 *represents gender;* 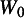 *and* 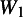 *denote the baseline body weight and final weight, respectively;* 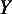 *is the weight gain and (b) NMA models:* 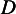 *represents study design (XY vs XZ);* 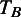 *and* 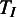 *denote the effects of baseline treatment* 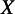 *and the intervention treatment (Y or Z);* 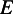 *is the difference in the effects between* 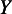 *and* 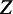 .

Figure 3a is a DAG for Lord’s Paradox, slightly modified from Pearl’s DAG.^19^ Each node represents a variable in the model; an arrow’s direction describes the relation between the two nodes. In Figure 3a, node G, gender, is a cause of baseline body weight ( 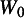 ) and final weight ( 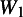 ), while 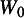 is a cause of 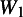 and weight gain ( 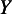 ), and 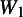 is a cause of 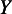 . The letter or number associated with an arrow denotes the strength of the path represented by the arrow and can be interpreted as a regression coefficient. For example, because on average, weight does not change for either males or females, the effect of 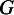 on 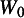 is equal to its effect on 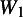 , which can be written as

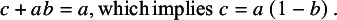



Statistician 1 considers the total effect of 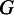 on 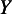 , the sum of three paths from 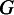 to 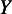 , 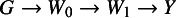 , 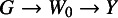 , and 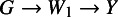 , so the total effect is 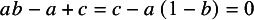 . Statistician 2, however, considers the conditional effect of 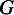 on 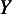 by blocking paths through 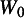 ; this conditional effect is 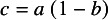 . The two statisticians have the same result if 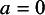 or 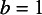 , i.e., (respectively) males and females do not differ in baseline body weight, or the regression of final weight on baseline weight has a coefficient of 1.

Figure 3b shows an analogous DAG for NMA. Node 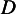 represents design (XY vs XZ), which influences the effects of both the baseline treatment 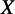 ( 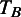 ) and the intervention treatment *Y* or *Z* ( 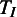 ). 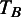 is a cause of 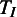 and of the difference in the effects of 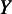 and 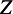 ( 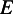 ), and 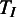 is a cause of 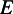 . Suppose the average relative treatment effect (difference between treatments) is the same in designs XY and XZ, so the effects of Y and Z relative to X do not differ, on average. Then the effect of 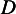 on 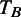 and 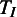 are the same, which implies


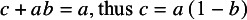



The CBM considers the total effect of 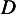 on 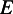 , the sum of three paths from 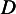 to 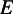 : 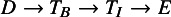 , 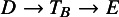 , and 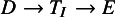 , so the total effect is 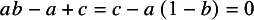 . The BM estimates the conditional effect of 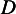 on 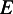 by blocking paths through 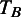 ; this conditional effect is 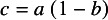 . The two models give the same result if 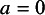 or 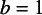 , i.e., (respectively) X has the same effect in designs XY and XZ, or the regression of the intervention treatment (Y or Z) on the baseline treatment (X) has a coefficient of 1.

Figure 3a shows that the *t*-test and ANCOVA give the same result if either the average baseline body weights are identical or the coefficient for regressing final body weight on baseline body weight is 1. In his 1967 article, FM Lord was more concerned about the latter, as this coefficient tended to be less than 1 because of natural fluctuations in body weights and measurement errors, causing imperfect correlation between two body weight measurements. In a NMA, even if the differences in the average treatment effects between X and Y and between X and Z are the same, the correlations (across studies) between treatment effects are unlikely to be 1, so the CBM and BM give different results.

## 7 Discussion

### 7.1 Comparing the two NMA models

When the baseline effects have similar distributions in the different trial designs, the contrast-based and BMs yield similar results. This is analogous to using the t-test or ANCOVA to analyze change scores from a randomized trial: both tests yield the same results because the treatment groups have similar baseline values.^20^ The key question, therefore, is which model is more appropriate when trial designs have different baseline effects.

Variation between trials in the effect modifiers of individual patients can lead to differences in baseline effects. Including study-level effect modifiers in the analysis may reduce unexplained variation between trials, but caution is still necessary. Moreover, some effect modifiers may be unknown or unavailable. Also, trials with similarly distributed effect modifiers may still have different baseline effects. If the baseline and CBM show substantially different results, this suggests that baseline effects are heterogeneous across trial designs. Caution is therefore needed in interpreting results from either model, as this heterogeneity may reflect an imbalance in the distribution of some effect modifiers, resulting in violation of the transitivity assumption. We may need to reassess the eligibility of individual trials against the prespecified criteria in the systematic review’s protocol.

Figure 3’s DAGs offer another perspective on the differences between the two models. The BM estimates the adjusted difference between Y and Z in the treatment effects conditional on the effect of X, while the CBM estimates the total, unadjusted difference. Whether the adjusted or unadjusted difference is more clinically meaningful is likely to be context specific. For instance, suppose the effect modifiers are distributed similarly in trials of different designs, while the effects of X are on average slightly higher in design XZ than in design XY. The BM may give a more precise estimate for the difference between Y and Z by adjusting for the heterogeneity in X’s effect. In contrast, if patients’ characteristics differ substantially in designs XZ and XY, neither of these two models can provide a definitive answer about the difference between Y and Z.

### 7.2 Different formulations of NMA models and selection of the baseline treatment

In our discussion of the two NMA models, the treatment X was included in every trial, so it was the natural candidate for both the baseline and reference treatment. However, it is rare for a treatment to be included in all trials, so our single-level formulation of the BM in Equations (10)–(12), with treatment contrasts as the unit of analysis, is not applicable to most NMAs. Nevertheless, we used this formulation to show the similarity between these NMA models and Lord’s Paradox. A more practical formulation of these NMA models uses treatment arms as the unit of analysis, in which case any treatment can be the reference or baseline treatment (if Model 2’s specification is used; see the Supplementary Material).

In a previous study, Shi and Tu^6^ used structural equation modeling to show that the CBM is a BM with the variance of the random study effects approaching infinity. This implies that results from the BM are not affected by the selection of reference treatment. There are, in fact, two versions of the BM that differ in their specification of random effects.^5^ ^,^ ^6^ The choice of a reference treatment affects the results in one specification but not the other. The Supplementary Material gives a more thorough technical discussion of the BM’s two specifications and of how the choice of the reference treatment affects their results differently.

Finally, although we used a specific NMA to demonstrate the analogy between Lord’s Paradox and the two NMA models, the conclusions and implications of this analogy extend beyond the specific NMA. For instance, we showed that the CBM uses observed treatment contrasts as outcomes, while the BM uses adjusted treatment contrasts. Thus, if the baseline treatment’s effect varies substantially across different designs of trials, these two models may yield different results. We refer readers to our previous article on bias propagation in NMA for simulations and a complex example in which results from these models can differ greatly.^21^

## 8 Conclusion

This article used Lord’s Paradox to provide a framework for comparing the CBM and BMs for NMA. These two models make different assumptions about the relationships between baseline and relative treatment effects. When they yield substantially different results, we need to be cautious in interpreting either model’s results.

## Supporting information

Tu and Hodges supplementary material Tu and Hodges supplementary material

## Data Availability

The R code for generating the example data used in this study is available in the Supplementary Material.

## Supplementary material

To view supplementary material for this article, please visit http://doi.org/10.1017/rsm.2025.10036.
