# Supplementary material for: Lord’s Paradox and two network meta-analysis models
Source: Res Synth Methods. 2025 Sep 18;17(1):111–22. doi: 10.1017/rsm.2025.10036 (PMC12823209; doi:10.1017/rsm.2025.10036)
Supplement: Tu and Hodges supplementary material [file S1759287925100367sup001.zip › Appendix to Lords Paradox paper 2025-03-12.pdf]

Appendix: On the baseline model's specification

Appendix to: "Lord's Paradox and Network Meta-analysis Models", by Yu-Kang Tu and James Hodges.

1 Introduction

This appendix considers two specifications for the baseline model that have appeared in the literature, one of which is used in the main paper. Sections 2 and 3 below show that for the specification used in the main paper, called Specification A below, the choice of reference treatment does not affect the analysis results, while for the other specification, Specification B, which is used in, e.g., White et al (2019), the choice of reference treatment *does* affect the analysis results. Section 4 argues briefly that Specification A should be preferred in network meta-analysis.

2 Notation with treatment 1 as the reference

Assume study $i, i = 1, \ldots, n$, includes treatments indexed by k, where $k \in \{1, \ldots, K\}$ and R_i is the subset of treatments included in study i. Assume treatment k in study i has an outcome y_{ik} on a continuous scale, modeled as $y_{ik} = \theta_{ik} + \epsilon_{ik}$, where $\epsilon_{ik} \sim N(0, se_{ik}^2)$ and the variance se_{ik}^2 is assumed known. The following concerns the further specification of θ_{ik}.

With treatment $k = 1$ as the reference treatment, model θ_{ik} as

$$\theta_{ik} = s_i + \delta_{i1k}, \tag{1}$$

where δ_{i1k} is the difference between treatment k and the reference and $s_i \sim N(\mu_1, \sigma_A^2)$, the latter being the baseline model's distinctive feature. In the model with heterogeneity, the K unknowns $\delta_{i1k}, k = 1, \ldots, K$, are modeled as

$$\boldsymbol{\delta}_i = (\delta_{i11}, \delta_{i12}, \ldots, \delta_{i1K}) \sim N_K(\boldsymbol{\mu}, \boldsymbol{\Sigma}), \tag{2}$$

where $\boldsymbol{\mu} = (0, \mu_{12}, \ldots, \mu_{1K})'$. As in White et al (2019), the specification in equations (1,2) includes in study i all K treatments even though only the subset R_i was actually oberved in study i. Further details of Specifications A and B differ, as follows.

2.1 Specification A

Specification A, called Model-2 in the main paper, is

$$\begin{aligned} y_{ik} &= s_i + \delta_{i1k} + \epsilon_{ik}, \quad \epsilon_{ik} \sim N(0, se_{ik}^2) \\ s_i &\sim N(\mu_1, \sigma_A^2), \quad \delta_{i1k} \sim N(d_{1k}, \tau^2), \quad d_{11} = 0 \end{aligned}$$

In terms of the notation defined above, this implies that

$$\boldsymbol{\Sigma} = \tau^2 \mathbf{I}_K, \tag{3}$$

where $\mathbf{I}_K$ is the K-dimensional identity matrix. (The results that follow also work if $\boldsymbol{\Sigma}$ is a compound symmetric covariance matrix.) Piepho et al (2024) introduced a similar specification (their equation 2) for a somewhat different purpose.

2.2 Specification B

Specification B differs from Specification A as follows:

$$\begin{aligned} y_{ik} &= s_i + U_{ik}\delta_{i1k} + \epsilon_{ik}, \quad \epsilon_{ik} \sim N(0, se_{ik}^2) \\ s_i &\sim N(\mu_1, \sigma_A^2), \quad U_{ik} = 1 \text{ if } k \neq 1 \text{ and } 0 \text{ if } k = 1, \quad \delta_{i1k} \sim N(d_{1k}, \tau^2), \quad d_{11} = 0 \end{aligned}$$

White et al (2019; model 3, Section 2.3.3) represent this by omitting the U_{ik} and requiring $\delta_{i11} \equiv 0$. Either way of writing Specification B implies that

$$\boldsymbol{\Sigma} = \left[\begin{array}{c|c} 0 & \mathbf{0}_{1\times(K-1)} \\ \hline \mathbf{0}_{(K-1)\times 1} & \boldsymbol{\Sigma}_{(-)} \end{array}\right], \tag{4}$$

where $\boldsymbol{\Sigma}_{(-)}$ is a $(K-1)\times(K-1)$ covariance matrix.

3 Now change the reference treatment

Rewrite $s_i \sim N(\mu_1, \sigma_A^2)$ as $s_i = \mu_1 + c_i$, where $c_i \sim N(0, \sigma_A^2)$, and rewrite

$$\boldsymbol{\delta}_i = \begin{bmatrix} \delta_{i11} \\ \delta_{i12} \\ \vdots \\ \delta_{i1K} \end{bmatrix} \sim N_K(\boldsymbol{\mu}, \boldsymbol{\Sigma}) \tag{5}$$

$$\text{as} \quad \boldsymbol{\delta}_i = \begin{bmatrix} 0 \\ d_{12} \\ \vdots \\ d_{1K} \end{bmatrix} + b_i, \quad \text{where} \quad b_i = \begin{bmatrix} b_{i1} \\ b_{i2} \\ \vdots \\ b_{iK} \end{bmatrix} \sim N_K(\mathbf{0}_K, \boldsymbol{\Sigma}). \tag{6}$$

Using equations (5, 6), combine equation (1) across treatments k to give

$$\boldsymbol{\theta}_i = \begin{bmatrix} \theta_{i1} \\ \theta_{i2} \\ \vdots \\ \theta_{iK} \end{bmatrix} = \mu_1 \mathbf{1}_K + \begin{bmatrix} 0 \\ d_{12} \\ \vdots \\ d_{1K} \end{bmatrix} + c_i \mathbf{1}_K + \begin{bmatrix} b_{i1} \\ b_{i2} \\ \vdots \\ b_{i1} \end{bmatrix}, \tag{7}$$

where $\mathbf{1}_K$ is a K-vector of 1's.

First, change the reference treatment in the fixed-effect part of the model, which is the first two terms in the right-hand side of (7):

$$\mu_1 \mathbf{1}_K + \begin{bmatrix} 0 \\ d_{12} \\ \vdots \\ d_{1K} \end{bmatrix} = \begin{bmatrix} \mu_1 \\ \mu_2 \\ \vdots \\ \mu_K \end{bmatrix}, \text{where } \mu_k = \mu_1 + d_{1k} \text{ for } k = 2, \ldots, K$$

$$= \mu_2 \mathbf{1}_K + \begin{bmatrix} d_{21} \\ 0 \\ \vdots \\ d_{2K} \end{bmatrix}, \text{where } d_{2k} = \mu_k - \mu_2 \text{ for } k \neq 2. \tag{8}$$

The difference between Specifications A and B affects the 3rd and 4th terms in the right-hand side of (7), as follows.

In Specification A, the 3rd and 4th terms are

$$c_i \mathbf{1}_K + \begin{bmatrix} b_{i1} \\ b_{i2} \\ \vdots \\ b_{iK} \end{bmatrix}, \quad \text{with cov}(\begin{bmatrix} b_{i1} \\ b_{i2} \\ \vdots \\ b_{iK} \end{bmatrix}) = \tau^2 \mathbf{I}_K,$$

$$\text{so} \quad \text{cov}(c_i \mathbf{1}_K + \begin{bmatrix} b_{i1} \\ b_{i2} \\ \vdots \\ b_{iK} \end{bmatrix}) = \sigma_A^2 \mathbf{1}_K \mathbf{1}_K' + \tau^2 \mathbf{I}_K. \tag{9}$$

Equation (9) is symmetric in the treatments and thus is unaffected when the reference treatment changes. Together with equation (8), this implies that changing the reference treatment does not affect the estimates of the random-effect variances, or the combined treatment differences, or the latter's standard errors.

In Specification B, the 3rd and 4th terms are

$$c_i \mathbf{1}_K + \begin{bmatrix} 0 \\ b_{i2} \\ \vdots \\ b_{iK} \end{bmatrix}, \quad \text{with cov}(\begin{bmatrix} 0 \\ b_{i2} \\ \vdots \\ b_{iK} \end{bmatrix}) = \left[\begin{array}{c|c} 0 & \mathbf{0}_{1\times(K-1)} \\ \hline \mathbf{0}_{(K-1)\times 1} & \mathbf{\Sigma}_{(-)} \end{array} \right]$$

$$\text{so} \qquad \text{cov}(b_i \mathbf{1}_K + \begin{bmatrix} 0 \\ b_{i2} \\ \vdots \\ b_{iK} \end{bmatrix}) = \left[\begin{array}{c|c} \sigma_A^2 & \sigma_A^2 \mathbf{1}_{1\times(K-1)} \\ \hline \sigma_A^2 \mathbf{1}_{(K-1)\times 1} & \mathbf{\Sigma}_{(-)} + \sigma_A^2 \mathbf{1}_{K-1} \mathbf{1}'_{K-1} \end{array} \right], \tag{10}$$

which is not symmetric in the treatments because the original reference treatment, treatment 1, is different from the other treatments. Thus the results of fitting this version of the baseline model depend on which treatment is the reference.

4 Specification A is better

Specification B might be considered tidier because in Specification A, only the reference treatment has two sources of heterogeneity, the model for s_i (which both Specifications use) and the random effect $b_{i1} \neq 0$. This apparent untidiness can be defined away if we say that the distribution of c_i in $s_i = \mu_1 + c_i$ does not describe how the s_i were generated but instead is simply a device to induce shrinkage in the estimated s_i. From this point of view, Secification A is tidier because it models heterogeneity of treatment effects the same way for all treatments, while Specification B does not.

Specification A has a clear advantage – the results of the analysis do not depend on the choice of reference treatment – which removes from the analysis an arbitrary element. On balance, this favors Specification A.

References

Piepho H-P, Madden LV, Williams ER (2024). The use of fixed study main effects in arm-based network meta-analysis. *Research Synthesis Methods*, 15(5):747-750. DOI: 10.1002/jrsm.1721

White IR, Turner RM, Karahalios A, Sananti G (2019). A comparison of arm-based and contrast-based models for network meta-analysis. *Statistics in Medicine*, 38:5197-5213, DOI: 10.1002/sim.8360
